# Ten-year outcomes of anterior cruciate ligament reconstruction with hamstring tendon autograft and femoral fixation with a cortico-cancellous screw suspension device

**DOI:** 10.1007/s00590-023-03740-6

**Published:** 2023-09-30

**Authors:** Elisa Senigagliesi, Luca Farinelli, Alberto Aquili, Pier Paolo Canè, Marco Fravisini, Antonio Pompilio Gigante

**Affiliations:** 1 Clinical Orthopaedics, Università Politecnica delle Marche, Via Tronto 10/a, 60020 Torrette di Ancona (AN), Italy; 2 Centro di Artroscopia e Chirurgia del Ginocchio, Clinica “Sol et Salus”, Rimini, Italy

**Keywords:** ACL reconstruction, Knee, Arthroscopy, Long-term follow up

## Abstract

**Purpose:**

To evaluate the clinical and radiographic outcomes of anterior cruciate (ACL) reconstruction at minimum 10-year follow-up.

**Methods:**

Ninety-three patients who underwent primary unilateral ACL reconstruction with hamstring tendon autograft, transtibial technique and femoral cortico-cancellous screw suspension device (Athrax, Leader Medica s.r.l) between 2010 and 2012 were retrospectively reviewed. Mean follow-up was 136 months. Evaluation was performed using the International Knee Documentation Committee score (IKDC), Knee injury and Osteoarthritis Outcome Score (KOOS), Lysholm Knee Score and Tegner Activity Level Scale. Incidence of OA was determined by comparing standard anteroposterior and lateral weightbearing radiographs of the ACL-reconstructed and contralateral knee. Osteoarthritis severity was graded according to the Kellgren–Lawrence (KL) score.

**Results:**

Median Tegner activity level was 6 (5–7). Lysholm and IKDC scores were 100 (95–100) and 90 (86–95), respectively, KOOS was 98 (95–100). Of ACL-reconstructed knees, 41 (50%) had radiographic OA, of which 6 (7.3%) had severe OA (KL III). Of the contralateral healthy knees, 28 (34.1%) had radiographic evidence of OA. Of these 22 (26.8%) and 6 (7.3%) patients had, respectively, KL-I and KL-II. 11 patients (11.8%) underwent subsequent knee surgery: 5 (5.4%) revisions, 3 (3.2%) meniscal surgeries, 2 (2.2%) other surgeries, 1 (1.1%) contralateral ACL reconstruction.

**Conclusions:**

The study demonstrates that ACL reconstruction with HT autograft and cortico-cancellous screw suspension device determines satisfying clinical results after 10 years of follow-up. From our cohort, a low rate of graft failure has been reported, even though almost 50% of patients present a knee OA greater or equal to grade II KL.

## Introduction

Anterior cruciate ligament (ACL) rupture is one of the most common knee injuries in the younger population. ACL reconstruction (ACLR) represents the treatment of choice especially in active patients that frequently desire to return to their pre-injury level of function and specific sport participation [1–3]. It has been widely reported that the short-term results of ACLR are good to excellent in most patients, with high rate of return to sports and low percentage of failures [4–6]. As regard to long-term outcomes, ACLR is thought to decrease the risk of osteoarthritis (OA) due to the re-established rotatory knee stability and subsequent decreased risk of meniscal and chondral damage [7]. However, several authors reported a high incidence of OA ranging between 21 and 50% after ACLR at 10- to 20-year follow-up [8, 9]. Moreover, a recent systematic review and meta-analysis found that transtibial ACLR resulted in a significantly greater incidence of OA compared with the anteromedial femoral tunnel drilling technique (53.7 vs 14.2%, *p *= 0.001) at 5- to 10-year follow-up [10]. Clinical outcomes of ACLR at minimum 10 years of follow-up have been assessed by several studies; however, these studies used bone–patellar tendon (BPT), allograft or Hamstring tendon (HT) combined to extra-articular reconstruction in all surgeries [11–19]. Furthermore, available data are limited when assessing outcomes beyond 10 years. The aim of the present study was therefore to assess the clinical outcomes after ACLR with transtibial technique and HT autograft with femoral cortico-cancellous screw suspension device at minimum 10-year follow-up by recording subjective outcomes (patient-reported outcome measures [PROMs]). The secondary aim of this retrospective study was to determine whether ACL-reconstructed knees with HT autograft have a greater incidence of degenerative changes compared with the contralateral non-reconstructed knee. We hypothesized that the outcomes of ACLR were satisfactory at 10-year follow-up and that incidence of OA was similar to that reported in literature.

## Materials and methods

This study was performed after approval of the institutional review board. The surgery records and medical charts of consecutive patients who underwent a unilateral primary ACL reconstruction between January 2010 and December 2012 at our institution were eligible for inclusion (*n* = 480). Inclusion criteria: age > 18 years old, ACL reconstruction with HT autograft, transtibial technique with cortico-cancellous screw suspension device as femoral fixation. Exclusion criteria: previous contralateral surgeries or trauma, posterior cruciate ligament injuries, medial and/or lateral collateral ligament injuries in the ipsilateral knee, concomitant osteotomy procedures, previous ipsilateral knee surgery, patellar and/or quadriceps tendon graft, other systems of femoral ACL graft fixation, Kellgren–Lawrence grade ≥ 2 in the ipsilateral or contralateral knee at the time of surgery. Eligible patients (*N *= 102) were contacted and invited to visit our outpatient clinic again. Nine patients could not be contacted or were lost to follow-up. This resulted in a total of 93 patients who were included for the overall analysis. When other concomitant and/or contralateral knee surgeries occurred, patients were not included for the assessment of PROMs or OA. Reconstruction was performed once the knee had recovered from the acute trauma of the ACL injury and patients had a pain-free knee, no extension deficit and at least 90° of knee flexion.

### Surgical technique

**Fig. 1:**
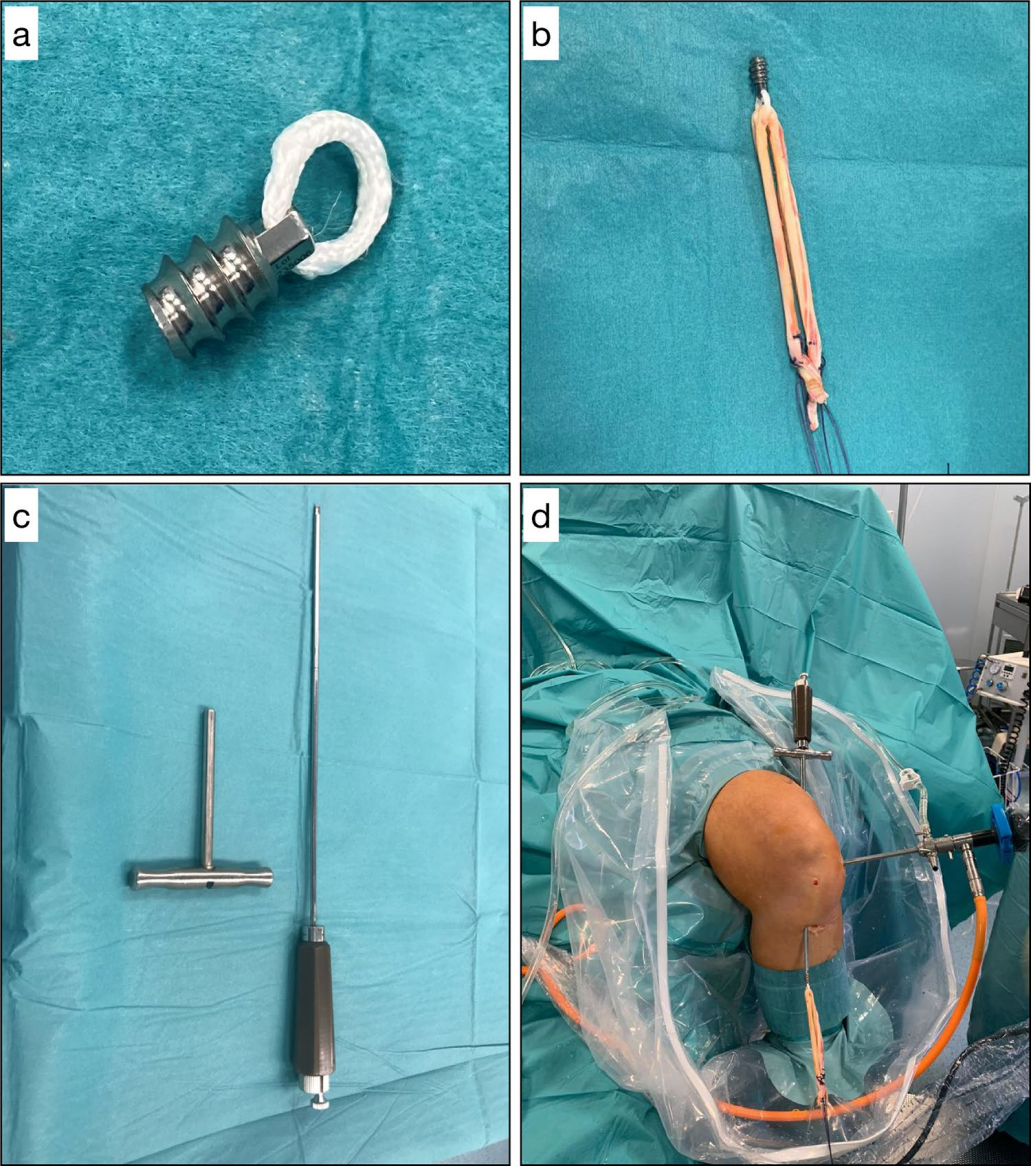
Athrax device (**a**), graft duplicated and inserted into the Athrax polyester eyelet (**b**), Athrax cannulated screwdriver (**c**), screwdriver advanced through the femoral and tibial tunnels at 90 degrees of knee flexion with the screw locked at its end (**d**)

**Fig. 2:**
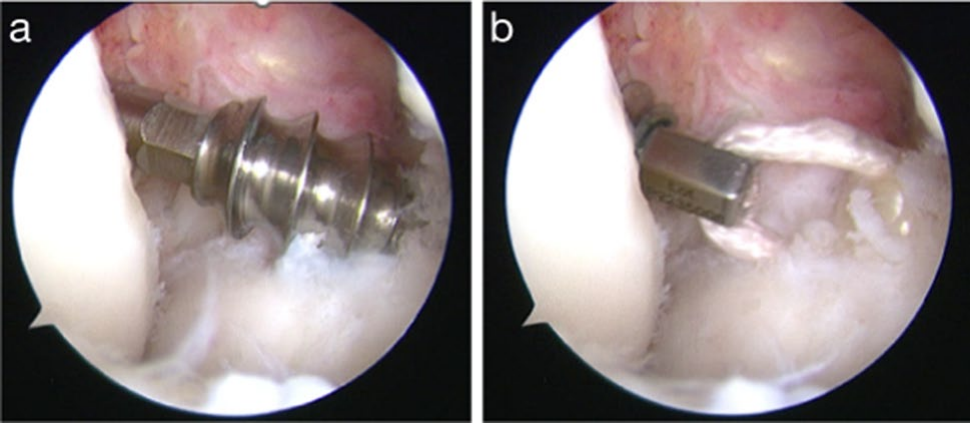
Arthroscopic view of the graft passing the tibial and femoral tunnels.

The following standardized surgical technique and postoperative rehabilitation protocol was adopted for all patients. Semitendinosus (ST) and gracilis (G) are harvested. The graft is inserted into the Athrax device polyester eyelet on the Arthrax screw (Leader Medica srl, Padua, Italy) and its ends are tubularized using a 2–0 Vicryl suture for each side (Fig. 1a–b). The graft is duplicated. Transtibial technique is used. A small skin incision is made at the exit point of the guide wire and the proper Athrax cannulated screwdriver (Fig. 1c) is inserted and advanced through the femoral and tibial tunnels while maintaining 90 degrees of knee flexion (Fig. 1d). The Athrax device screw with the graft loaded is then attached to the appropriate screwdriver. The Athrax screw is locked at the screwdriver and passed back through the tunnels until meeting the external femoral cortex. The Athrax screw is then screwed counterclockwise to be securely fixed into the femoral cortex. Figure 2a–b shows the arthroscopic view of the graft passing the tunnels. The screwdriver is left attached while proceeding to the tibial fixation. A unicortical cannulated poly lactic-co-glycolic acid (PLGA) interference screw is advanced through the tibial tunnel. The graft is tensioned at about 20 degrees of knee flexion. The Athrax screw device allows the surgeon to perform a graft retensioning even after the tibial fixation. To the best of our knowledge, this graft femoral fixation technique has never been described before. Rehabilitation program is initiated immediately with active range-of-motion exercises mainly focusing on restoring full extension. If a meniscal suture was performed, flexion was limited to 90 degrees for 4 weeks. The patient is mobilized partially weightbearing (15–20 kg) for 4 weeks. Full weightbearing and free range of motion are allowed thereafter as tolerated and no additional meniscus injury provided.

### Outcomes

Graft ruptures, contralateral ACL tears and reoperations were assessed. Subjective outcomes were measured using the following validated patient-reported outcome scores at follow-up: subjective IKDC knee form, Knee injury and Osteoarthritis Outcome Score (KOOS), Lysholm Knee Score and Tegner Activity Level Scale. Incidence of OA was determined by comparing standard anteroposterior and lateral weightbearing radiographs of the ACL-reconstructed and contralateral (healthy) knee. Osteoarthritis severity was graded according to the Kellgren–Lawrence (KL) grade. All radiographs were independently graded by 2 senior surgeons. In case of any controversy, agreement was reached by discussion.

### Statistical analysis

Data were collected and managed using Excel (Microsoft, Redmond, WA, USA). Statistical analyses were performed using the XLSTAT statistical software packages (Addinsoft LLC, Paris, France). Categorical variables were expressed using numbers and percentages (%). Normality of the distribution was evaluated using Shapiro–Wilk and Kolmogorov–Smirnov tests. None of the data were normally distributed; therefore, nonparametric statistical tests were used, and data were presented in median (interquartile range). Mann–Whitney* U* tests were used for continuous variables and chi-square tests for categorical values. Analysis of covariance (ANCOVA) was performed to examine the independent associations between radiographic evidence (Kellgren–Lawrence) of OA and explanatory variables as age at time of surgery, body mass index, baseline Tegner activity level, timing of surgery, medial meniscus injuries and lateral meniscus injuries. The significance level was set at *P* = 0.05.

## Results

### Study group

Overall, 93 consecutive participants undergoing ACL reconstruction who met the inclusion and exclusion criteria formed the study group. The mean age at surgery was 30.2 years, and 68 patients (73.1%) had their reconstruction performed within 6 months of the injury. There were 48 left-sided and 45 right-sided reconstructions. Mean follow-up was 136 months (range 120–153 months). Reoperations occurred in 10 patients (10.8%). Specifically, 3 (3.2%) patients underwent to partial meniscectomies following a new knee trauma occurred during follow-up period. Five (5.4%) patients underwent to ACL revision due to graft failure after new trauma. One patient (1.1%) underwent to posterior cruciate ligament injury following car accident. One patient (1.1%) underwent to knee arthroscopy due to stiffness. One patient (1.1%) underwent ACL reconstruction in the contralateral knee. Baseline characteristics of study group are summarized in Table 1.

**Table 1:** Baseline patient characteristics

	Available at follow-up (*n* = 93)
Age, years	
At surgery	28 (23–38)
At follow-up	39 (34–49)
Follow-up, months	133 (132–144)
Male sex	66 (71%)
BMI	23.9 (2.7) [18–37]
Timing surgery,* N* (%)	
0–3 months	40 (43.0%)
3–6 months	28 (30.1%)
> 6 months	25 (26.9%)
Meniscal tears	
MM tear	19 (20.4%)
LM tear	12 (12.9%)
MM + LM tear	4 (4.3%)
LET	2 (2.2%)
Graft rupture	5 (5.4%)
Controlateral ACL injury	1 (1.1%)
Reoperation	
Meniscal surgery	3 (3.2%)
Revision surgery	5 (5.4%)
Others	2 (2.2%)

Values are displayed as median (interquartile range) or No (%)

* ACL* anterior cruciate ligament;* BMI* body mass index;* LM* lateral meniscus;* MM* medial meniscus;* LET* lateral extra-articular tenodesis.

### Patient-reported outcome scores and radiographic outcomes

Patients with an intact ACL graft had a median Tegner activity level of 6 (5–7) (Table 2). They reported excellent PROMs, with a median Lysholm score of 100 (95–100), a median subjective IKDC score of 90 (86–95) and a KOOS of 98 (95–100). Of ACL-reconstructed knees, 41 (50%) had no signs of radiographic OA, and 41 (50%) had radiographic OA, of which 6 (7.3%) had severe OA (KL III). Of the contralateral healthy knees, 54 (65.9%) had no signs of OA, and 28 (34.1%) had radiographic evidence of OA. Of these 22 (26.8%) and 6 (7.3%) patients had, respectively, KL-I and KL-II of OA. The grade of knee OA between operated and contralateral knee differ significantly at follow-up (*p* = 0.016) (Table 3). From analysis of covariance (ANCOVA), it has been found that age at time of surgery should be associated with radiographic evidence of knee OA after minimum 10 year of follow-up (odds ratio 1.14–95% CI 1.15–1.74, *p* = 0.001). Baseline Tegner activity level, timing of surgery, medial and/or lateral meniscus injuries and BMI were not associated to radiographic sign of OA (*p* = 0.523, *p*=0.489, *p* = 0.118, *p* = 0.890, *p* = 0.803). Significant difference was reported between Tegner activity level score at the time of surgery and after the follow-up period (*p* = 0.03).

**Table 2:** PROMs: Patient report outcomes measurements

	Available at follow-up (*n* = 82) *	
PROMs		
KOOS	98 (95–100)	
IKDC	90 (86–95)	
LYSHOLM knee score	100 (95–100)	
Tegner Activity level score		
At surgery	7 (5–7)	*p* value 0.03
At follow-up	6 (5–7)	

*11 patients were excluded to overall analysis due to subsequent meniscal surgery (*n* = 3), graft failure (*n* = 5), contralateral ACL injury (*n* = 1), others knee surgery (*n* = 2)

*IKDC* International Knee Documentation Committee;* KOOS* knee injury and osteoarthritis outcome score

**Table 3:** Radiographic evidence of osteoarthritis in the operated and contralateral knee

	At follow-up (*n*=82)	
KL grade	ACL side	Opposite side
No sign of OA	41 (50%)	54 (65.9%)
KL-I	22 (26.8%)	22 (26.8%)
KL-II	13 (15.9%)	6 (7.3%)
KL-III	6 (7.3%)	0
*p* value	**0.016**	

*KL* Kellgren–Lawrence;* OA* osteoarthritis

## Discussion

The most important findings of the present study were that a relatively low graft rupture rate (5.4%) was reported after ACLR with transtibial technique, HT autograft and femoral cortico-cancellous screw suspension device. Although the Tegner activity level score significantly decreased compared to pre-surgery values, clinical and subjective outcome measures demonstrated that most patients were satisfied with the surgical outcome and quality of life. However, 41 (50%) patients had radiographic signs of OA in the ACL-reconstructed knee.

Analyzing the current literature, Leiter et al. [20] reported a graft rupture rate of 9% with HT graft reconstruction at 14.6 years of follow-up. Asik et al. [12] found a graft failure rate of HT of 1.5% in a cohort of 271 patients. However, the mean follow-up period was 82 (minimum 48 and maximum 108) months. A recent systematic review and meta-analysis that compares the clinical results between HT and patellar tendon reported a failure rate of HT graft from 6.3% to 18% at minimum 10 years of follow-up [21]. Our results showed a lower rate of graft failure (5.4%) compared to existing literature [22]. This difference could be explained by the fact that the mean age of their cohort at the time of surgery was 25 years, whereas the median age in our cohort was 28 (23–38) years at the time of surgery and several studies have shown that the failure rate of ACLR is higher in younger patients [23, 24]. Moreover, the decreased Tegner activity score at time of follow-up compared to pre-surgery levels could minimize the risk of graft failure after ACLR.

Our results were in line with the current literature concerning the incidence and severity of OA after ACLR. A recent systematic review and meta-analysis reported a high incidence of OA in ACL-reconstructed knee with HT graft after 20 years of follow-up [25]. Specifically, a grade of knee OA greater than or equal to 2 according to the Kellgren–Lawrence classification was found between 40% and 66% of cases. However, it is crucial to consider that the studies included in that review are dated to the 1990s and the current standard of practice could be more accurate in terms of tunnel preparation and femoral tunnel placement. However, these data were similar to those reported by Sollberger et al. [21]. In their systematic review, they reported a rate of knee OA ≥ 2 (KL classification) in 32–55.2% of cases after 10 years of follow-up. Although there is a large variation in the incidence of OA in ACL-reconstructed knees at long-term follow-up, meniscectomy has been shown to be a very strong predictor of OA [26]. However, these data were not confirmed by our study. A possible explanation could be that meniscal lesions have been found in a relatively low percentage of patients of our cohort; therefore, the sample size could not be enough to detect significance from multivariate analysis.

On the other hand, from our results, it has been reported that age at time of surgery represent a significant risk of developing knee OA after minimum 10 years of follow-up (odds ratio 1.14–95% CI 1.15–1.74, *p* = 0.001). Our results were in line with several studies. Hence, Curado et al reported that meniscectomy and age at time of surgery represent main risks for the development of subsequent knee OA [27].

The present study has several limitations that warrant disclosures. First of all, this is a retrospective study, and no control group was assessed. Then, even though consecutive patients have been recruited a selection bias may be present. Moreover, the limited sample size could make the study underpowered for multivariate analysis. Nine patients (8.8%) could not be contacted and were lost at follow-up.

## Conclusion

The findings in the present study show that ACLR with HT graft and cortico-cancellous screw suspension device determined satisfied clinical results after 10 years of follow-up. From our cohort, a low rate of graft failure has been reported, even though almost 50% of patients present a knee OA greater or equal to grade II KL.

## Data Availability

The datasets used and/or analyzed during the current study are available from the corresponding author on reasonable request.
